# Reasons for Exclusion of Apparently Healthy Mature Adult and Senior Dogs From a Clinical Trial

**DOI:** 10.3389/fvets.2021.651698

**Published:** 2021-06-02

**Authors:** Nicole H. Gibbs, Hannah Michalski, Daniel E. L. Promislow, Matt Kaeberlein, Kate E. Creevy

**Affiliations:** ^1^Department of Small Animal Clinical Sciences, College of Veterinary Medicine and Biomedical Sciences, Texas A&M University, College Station, TX, United States; ^2^Department of Laboratory Medicine & Pathology, University of Washington School of Medicine, Seattle, WA, United States; ^3^Department of Biology, University of Washington, Seattle, WA, United States

**Keywords:** healthy cohort effect, exclusion criteria, aging, enrollment, older dogs

## Abstract

**Background:** Interventional clinical trials intended to maintain health in aging dogs are unusual and require particular attention to exclusion criteria.

**Objectives:** To describe reasons for exclusion when a mature adult and senior canine population with normal health status was sought.

**Animals:** Fifty six companion dogs nominated for a randomized controlled trial (RCT).

**Procedures:** Exclusions occurred within Stage 1 (S1): owner-provided survey information; Stage 2 (S2): medical records review; and Stage 3 (S3): screening examination and within Owner, Dog, or Other factor categories.

**Results:** Of 56 nominated dogs, 39 were excluded at S1 (*n* = 19), S2 (*n* = 5), and S3 (*n* = 15), respectively. Dogs were excluded for Owner (*n* = 4), Dog (*n* = 27), Other (*n* = 6), and concurrent (Owner + Dog; *n* = 2) factors. The most common exclusion period was S1 (*n* = 19), with weight outside the target range being the most common exclusion factor in that stage (*n* = 10). Heart murmurs were the second most common exclusion factor (S1: *n* = 1; S3: *n* = 5); suspected or confirmed systemic illness was third most common (S1: *n* = 2; S2: *n* = 3; S3: *n* = 2). Among dogs who passed S1 and S2 screening (*n* = 32), 15 dogs (48%) were excluded at S3, for heart murmur > grade II/VI (*n* = 5), cardiac arrhythmias (*n* = 2), and clinicopathologic abnormalities (*n* = 2).

**Conclusions and Clinical Relevance:** Dogs nominated for a clinical trial for healthy mature adult and senior dogs were excluded for size, previous diagnoses, and newly discovered cardiac abnormalities. For future interventions in mature adult and senior dogs of normal health status, it is important to define expected age-related abnormalities to ensure that meaningful exclusion criteria are used.

## Introduction

Studies of pharmaceutical interventions to maintain health, rather than to treat disease, in healthy mature adult dogs are unusual. Mature adult dogs are defined by the American Animal Hospital Association as those who have completed physical and social maturation and have not yet reached the last 25% percent of size-based expected lifespan; dogs within the last 25% of size-based expected lifespan are considered seniors (1, 2). Most clinical trials investigate drugs intended to treat or cure a specific disease; animals are enrolled based on the presence of that specific disease (3–5). Interventional clinical trials that commonly recruit healthy dogs include studies of vaccine efficacy (6–8), and anesthetic or analgesic protocols (9–11). Many such studies that have reported findings from healthy dogs often have considered a subject healthy upon enrollment if it had an unremarkable physical examination and its owner perceived it as healthy, without a more extensive diagnostic work-up (6, 8–11). Vaccine efficacy studies including healthy dogs are often performed in shelters because of the large number of dogs available (8, 12, 13). However, dogs presenting to shelters are frequently young and/or have unknown health backgrounds; the apparently healthy dogs reported in such studies seldom represent the full spectrum of age and/or may have undiagnosed ailments. Studies of anesthetic/analgesic protocols often recruit subjects undergoing elective sterilization surgery, who are also therefore likely to be young (9–11). By contrast, research into diagnostic techniques identifies “cases” who meet the gold standard of diagnosis for a particular condition; the “control” group is defined primarily by not having that disease (14–16). Control subjects are seldom extensively investigated for global health, but undergo sufficient diagnostic investigation to determine that they are free of a specific disease, or disease within a single organ system (17–19).

An exception is seen in Hall et al. (14). This study evaluated symmetric dimethylarginine in dogs with chronic kidney disease. The investigators had many objective criteria for exclusion of control dogs, including absence of historic systemic illness; normal annual physical examination, complete blood count, serum biochemical analyses, urinalysis, and fecal examination for parasites; and four sequential normal results for serum creatinine concentration and GFR testing by iohexol clearance over a 6-month period. However, it is important to note that these dogs came from a research dog colony and were selected retrospectively. This allowed the investigators to identify healthy dogs from a large pool of dogs whose health status had been extensively documented. In prospective clinical trials of client-owned mature adult and senior dogs, such as the authors' recent clinical trial of rapamycin (20), prospectively recruited dogs are unlikely to have such extensive documentation of prior and recent health status, and are likely to exhibit various mild abnormalities and reductions in function that dogs acquire through the normative aging process.

Mature adult and senior dogs are often perceived as healthy even with mild abnormalities in bloodwork or common mild illnesses (21). Willems et al. (21) investigated the results of physical examination and laboratory tests in mature adult and senior dogs that were judged by their owners to be healthy. Common abnormal findings included overweight body condition, heart murmurs and mild increases in liver enzyme activity; it was not clear whether these liver enzyme changes represented occult disease or merely indicated the need for age-based or subject-based reference intervals (22). It is unclear how such subclinical findings should be addressed when considering a dog for inclusion in a clinical trial targeting normally aging mature adult and senior dogs.

Rapamycin is an immunosuppressive drug used in human medicine (23, 24). Recent research has shown benefits of sub-immunosuppressive doses of rapamycin in invertebrates and mice (25, 26), including lengthened lifespan (27, 28) and protection against multiple age-associated health outcomes, including reduced cancer incidence (29, 30), maintenance of cognitive function (31, 32), rejuvenation of oral health (33, 34), improved kidney (35), ovarian function (36), restoration of the aged immune system (37), and cardiac function improvement (38–40).

Recently completed and ongoing clinical trials of rapamycin in companion dogs seek to determine the benefits of rapamycin, including extending the healthy lifespan of dogs (20). A small randomized controlled trial (RCT) investigated the safety and potential benefits of low-dose rapamycin in mature adult and senior (>6 years of age) medium to large (18–36 kg) companion dogs over a 10-week period (20, 41). Low-dose rapamycin refers to sub-immunosuppressive dosages. In this study, the two treatment groups received 0.05 mg/kg three times per week and 0.1 mg/kg three times per week, which is lower than doses expected to cause immune suppression in dogs (42, 43). Participant dogs were required to be healthy at the time of enrollment based on history (dogs with active diagnosese, or currently receiving medications were excluded), normal physical examination, and results within reference intervals for all parameters on baseline echocardiography, complete blood count, chemistry profile and urinalysis (20, 41). Results revealed no significant difference in adverse events between treated and placebo dogs, and improvement in echocardiography parameters in rapamycin-treated dogs (20). These findings prompted another rapamycin RCT in a similar demographic group of companion dogs over a year-long period. The same strict exclusion criteria for health were used, and it became clear that the meaning of “healthy” for mature adult and senior dogs requires clarification.

Because few canine studies have comprehensively described the meaning of “healthy” at the time of enrollment of normal or control populations, and because many studies involving healthy dogs happen to occur among younger dogs, there is a lack of a standard description of what constitutes a healthy mature adult or senior dog. Absence of any detectable abnormality is unlikely in this group (21, 44, 45). Similarly, there is a lack of robust actuarial description of common diseases in the aging canine population that could lead to evidence-based construction of age-related exclusion criteria. The phenomenon of “healthy cohort bias” exists when the study population is in better health than the target population and is well-documented in human clinical trials (46, 47). To avoid healthy cohort bias in an interventional clinical trial for healthy mature adult and senior dogs, it may be more appropriate to seek dogs with typical health for their age, or to define common expected abnormalities that will not be used as exclusion criteria. The purpose of the present study was to describe reasons for exclusion of mature adult to senior, medium to large companion dogs from a RCT of a drug targeted for use in healthy dogs.

## Methods

A recently completed placebo-controlled, double-blind RCT of rapamycin conducted at Texas A&M University (TAMU) College of Veterinary Medicine & Biomedical Sciences (CVMBS) included 6 months of treatment (low-dose rapamycin or placebo) and 6 months of post-treatment monitoring. Dogs between the ages of 6 and 10 years old that weighed between 40 and 80 pounds (18–36 kg) were enrolled from May 2018 through February 2019. This age and weight range was chosen to target this intervention at mature adult and senior dogs (1, 2). Parameters assessed for inclusion included history, physical examination, hematology, chemistry, urinalysis, blood pressure, ECG, and echocardiogram, with normal results required for all. Inclusion and exclusion criteria and all study protocols were defined in advance, and all procedures for this study were reviewed and approved by the TAMU Institutional Animal Care and Use Committee (IACUC 2017-0125 CA). Because dog owners provided information about their dogs in the home environment, a determination of Human Subjects Research (HSR) was sought from the TAMU Institutional Review Board (IRB) and the study was found not to be HSR.

The enrollment process had three stages: owner survey, review of referring veterinarian records, and an initial examination. This examination functioned as both a screening and baseline examination. If no exclusionary findings were revealed and the dog was enrolled, findings from this examination were considered baseline data for the RCT. If the owner became unresponsive to email or follow-up scheduling, then the dog was excluded at whichever stage was incomplete. The excluded dogs were classified by the stage at which they were excluded, as well as whether Owner, Dog, or Other factors led to exclusion.

Stage 1 was the initial owner survey. Interested owners were asked to complete a survey that included basic information about the dog and questions determining willingness to participate. Information gathered in this survey included the dog's age and weight, temperament of the dog, dog's ability to take oral medications, and previous medical history. Previous medical history provided by the owner included vaccination history, evaluation of consistent heartworm prevention administration, and whether the dog was currently healthy. Owner factors that led to exclusion in this stage included owners becoming unresponsive or indicating they were unable to attend four appointments at TAMU's Veterinary Medical Teaching Hospital (VMTH) over the course of the year. Dog factors that led to exclusion in this stage included age outside the target range (i.e., <6 or >10 years old), weight outside the target range (i.e., <40 lb or >80 lb), inability to take oral medications, or lapsed vaccination and heartworm preventive history. Other factors that led to exclusion included owner requesting guarantee of receiving rapamycin instead of placebo, as well as owner notifying the study team of any systemic illness or cardiac abnormality in the dog. Owner requesting a guarantee of treatment (vs. placebo) assignment was considered an Other factor because it may have resulted from inadequate explanatory information provided by the study team. Owner description of prior illness was considered an Other factor because it was reported by the owner but not confirmed through any medical record or consultation. If the dog and owner met the study criteria, then medical records from the referring veterinarian were requested.

Stage 2 included receiving and reviewing the records from the dog's primary veterinarian. The records were reviewed by a board-certified small animal internist (KEC). All available records were reviewed and a minimum of the most recent 3 years of records were required for consideration. Owner factors that led to exclusion included inability to obtain and provide the dog's previous veterinary medical record after three attempts. Once records were received, Dog factors that led to exclusion included age outside the target range (i.e., <6 or >10 years old), weight outside the target range (i.e., <40 lb or >80 lb), history, physical exam and/or clinical pathology findings suggestive of systemic illness, thyroid panel results suggestive of hypothyroidism, documented hypertension (i.e., systolic blood pressure >160 mmHg), cardiac concerns (detailed below), or notes on poor temperament of the dog in the veterinary setting. Cardiac concerns documented in the medical record that led to exclusion included positive heartworm test or absent history of heartworm preventive, cardiac arrhythmia other than sinus arrhythmia, heart murmur > grade II/VI, and diagnosed cardiac disease other than Stage B1 chronic valvular disease (CVD). Stage B1 CVD refers to asymptomatic patients that have no radiographic or echocardiographic evidence of cardiac remodeling (48). No Other factors were identified in Stage 2. Dogs not excluded in Stage 2 were asked to continue to Stage 3 of enrollment.

After review of veterinary records in Stage 2, the owner and dog were asked to come to an initial appointment at the TAMU VMTH for Stage 3 of the enrollment process. During the initial appointment, the owners were given information about the study and rapamycin and offered time to ask questions. Informed Owner Consent for participation was obtained before the screening/baseline examination procedures were performed. A physical examination was performed by a board-certified small animal internist (KEC). In addition, cardiac auscultation, electrocardiogram and echocardiogram were performed by a board-certified veterinary cardiologist. The board-certified veterinary cardiologist confirmed any heart murmurs auscultated on physical examination by the internist to determine whether or not that dog should be included or excluded from the study. In addition, if an apparent arrhythmia was ausculted, an ECG was used to confirm this finding. Blood and urine were collected for a heartworm test, complete blood count, chemistry panel, urinalysis, and total T4 concentration. If total T4 was low, free T4 and TSH were additionally submitted. Indirect systolic blood pressure was measured using Doppler (49). Owner factors that led to exclusion in Stage 3 included inability to schedule the screening/baseline appointment, or the owner's decision not to provide informed consent to continue with the study. Dog factors that led to exclusion in Stage 3 included physical exam or clinical pathology findings suggestive of systemic illness, thyroid panel results suggestive of hypothyroidism, hypertension (i.e., systolic blood pressure >160 mmHg), positive heartworm test, cardiac arrhythmia other than sinus arrhythmia present on auscultation or electrocardiogram, heart murmur > grade II/VI, cardiac disease evident on echocardiogram other than Stage B1 CVD, limited/poor echocardiographic scan window, or poor temperament during the appointment. Given that we were interested in the potential of rapamycin to mitigate normal age-related decline in heart function, the dogs were excluded if they had chronic valvular disease that had advanced to Stage B2. Unlike Stage B1 with no evidence of cardiac remodeling on imaging, Stage B2 refers to asymptomatic patients that have hemodynamically significant valve regurgitation, as evidenced by radiographic or echocardiographic findings of left-sided heart enlargement (48). Additionally, it is recommended to start pimobendan therapy in dogs with Stage B2 CVD to increase cardiac contractility and decrease the size of the heart; we did not want to withhold this recommendation from enrolled dogs or confound potential effects of pimobendan and rapamycin (48). Other factors that led to exclusion in Stage 3 included concurrent enrollment of another dog from the same household in this study; only one dog from any household could be enrolled to ensure accuracy of treatment and maintenance of owner blinding. If dogs were not excluded during Stage 3 due to Owner, Dog, or Other factors, then the dogs were enrolled in the year-long rapamycin RCT.

For data analysis, the excluded dogs were classified first by the stage (Stage 1, Stage 2, or Stage 3) at which they were excluded, and then by the factor category (Owner, Dog, or Other) for which they were excluded. After classification by stage and factor category, the excluded dogs were grouped into subcategories based on the exact reason for exclusion. For the Owner factors, an exclusion criterion present in all Stages was lack of response to follow-up communication. The other subcategories for Owner factors that caused exclusion were inability to attend four appointments at the TAMU VMTH (Stage 1), inability to comply with appointment or medication schedule (Stage 2), and inability to schedule initial appointment (Stage 3). Dog factors that led to exclusion from the clinical trial are listed by stage in Table 1. The number of dogs excluded by stage, factors, and subcategory were totaled to determine the common factors that affect enrollment of apparently healthy mature adult and senior companion dogs in a clinical trial.

**Table 1:** Specific reasons for exclusion in the Dog factor category (*n* = 29) during each stage. Stage 1 had six different subcategories that could have led to exclusion due to Dog factors, whereas, Stage 2 and Stage 3 each had 10 subcategories.

**Stage 1 Dog factors**	**Stage 2 Dog factors**	**Stage 3 Dog factors**
Not between 6 and 10 years of age (*n* = 2)	Not between 6 and 10 years of age (*n* = 0)	Heart murmur graded III/VI or louder (*n* = 5)
Not between 40 and 80 lb (18-36 kg) (*n* = 10)	Not between 40 and 80 lb (18–36 kg) (*n* = 2)	Limited or poor echocardiography scan window (*n* = 1)
Not cooperative for physical exam, blood pressure, phlebotomy, and/or echocardiography (*n* = 0)	History or physical examination findings suggestive of systemic illness (*n* = 2)	Physical exam findings suggestive of systemic illness (*n* = 0)
	Clinical pathology findings suggestive of systemic illness (*n* = 0)	Clinical pathology findings suggestive of systemic illness (*n* = 2)
	Thyroid panel results suggestive of hypothyroidism (*n* = 1)	Thyroid panel results suggestive of hypothyroidism (*n* = 0)
Not able to take oral medications for 6 months (*n* = 0)	Hypertension (SBP > 160 mmHg) (*n* = 0)	Hypertension (SBP > 160 mmHg)
Not currently receiving regular monthly heartworm preventative (*n* = 0)	Positive heartworm test or absent history of heartworm preventive treatment (*n* = 0)	Positive heartworm test (*n* = 0)
Not vaccinated as recommended by AAHA (*n* = 0)	Cardiac arrhythmia other than sinus arrhythmia (*n* = 0)	Cardiac arrhythmia other than sinus arrhythmia (*n* = 2)
	Cardiac disease other than Stage B1 CVD (*n* = 0)	Cardiac disease evident on echocardiogram other than Stage B1 CVD (*n* = 0)
	Not cooperative for physical exam, blood pressure, phlebotomy, and/or echocardiography (*n* = 0)	Not cooperative for physical exam, blood pressure, phlebotomy, and/or echocardiography (*n* = 2)

## Results

**Figure 1:**
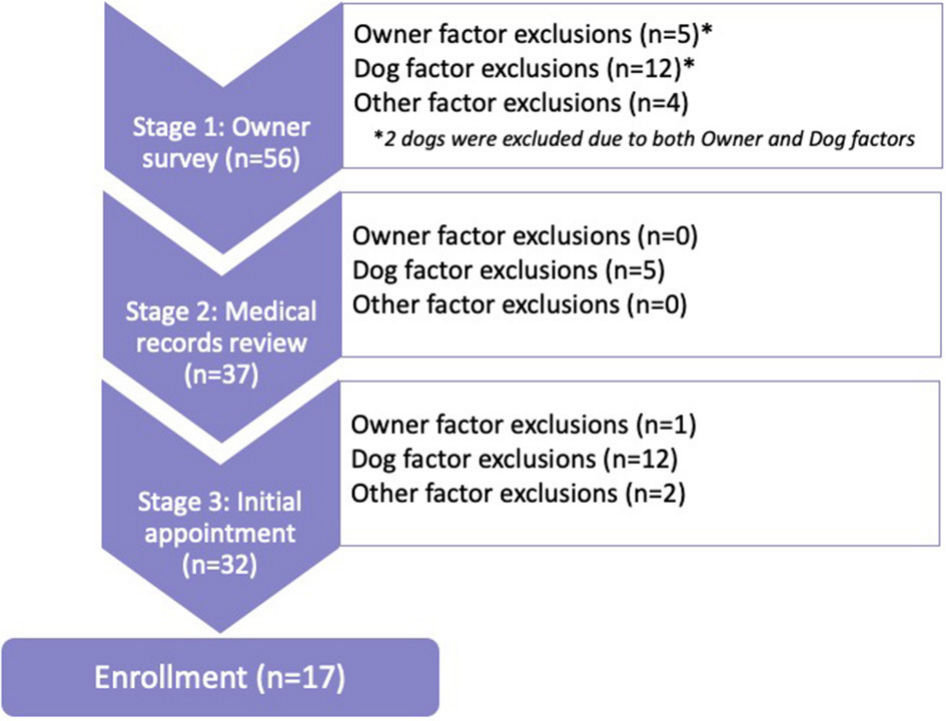
Total exclusions (*n* = 39) showing stages and factors.

Fifty-six owners contacted the study expressing interest in their dogs being considered for the clinical trial. Thirty-nine dogs (71%) were excluded during the enrollment process and 17 dogs were enrolled (Figure 1).

Stage 1 was the most common exclusion point (*n* = 19), followed by Stage 3 (*n* = 15), and Stage 2 (*n* = 5). Exclusion due to Dog factors (*n* = 29) far outweighed exclusion due to Owner factors (*n* = 6) or Other factors (*n* = 6). Two dogs were excluded for both Dog and Owner factors during Stage 1; both dogs weighed over 80 pounds and both owners were unable to attend four appointments at the TAMU VMTH throughout the year.

Of the four dogs excluded due to Owner factors alone, three dogs were excluded in Stage 1 because the owners were unable to attend the required appointments at TAMU VMTH. One dog was excluded in Stage 3 because the owner was unable to schedule an initial appointment. Of the six dogs excluded due to Other factors alone, four were excluded in Stage 1 and two were excluded in Stage 3. Two owners in Stage 1 wanted a guarantee of receiving rapamycin for their dogs, which led to exclusion. Both dogs excluded for Other factors in Stage 3 had housemate dogs qualify. This study did not allow multiple dogs from the same household to be enrolled. The other two dogs excluded due to Other factors were excluded in Stage 1 due to owner-reported heart murmur or illness not verified by medical records review.

**Figure 2:**
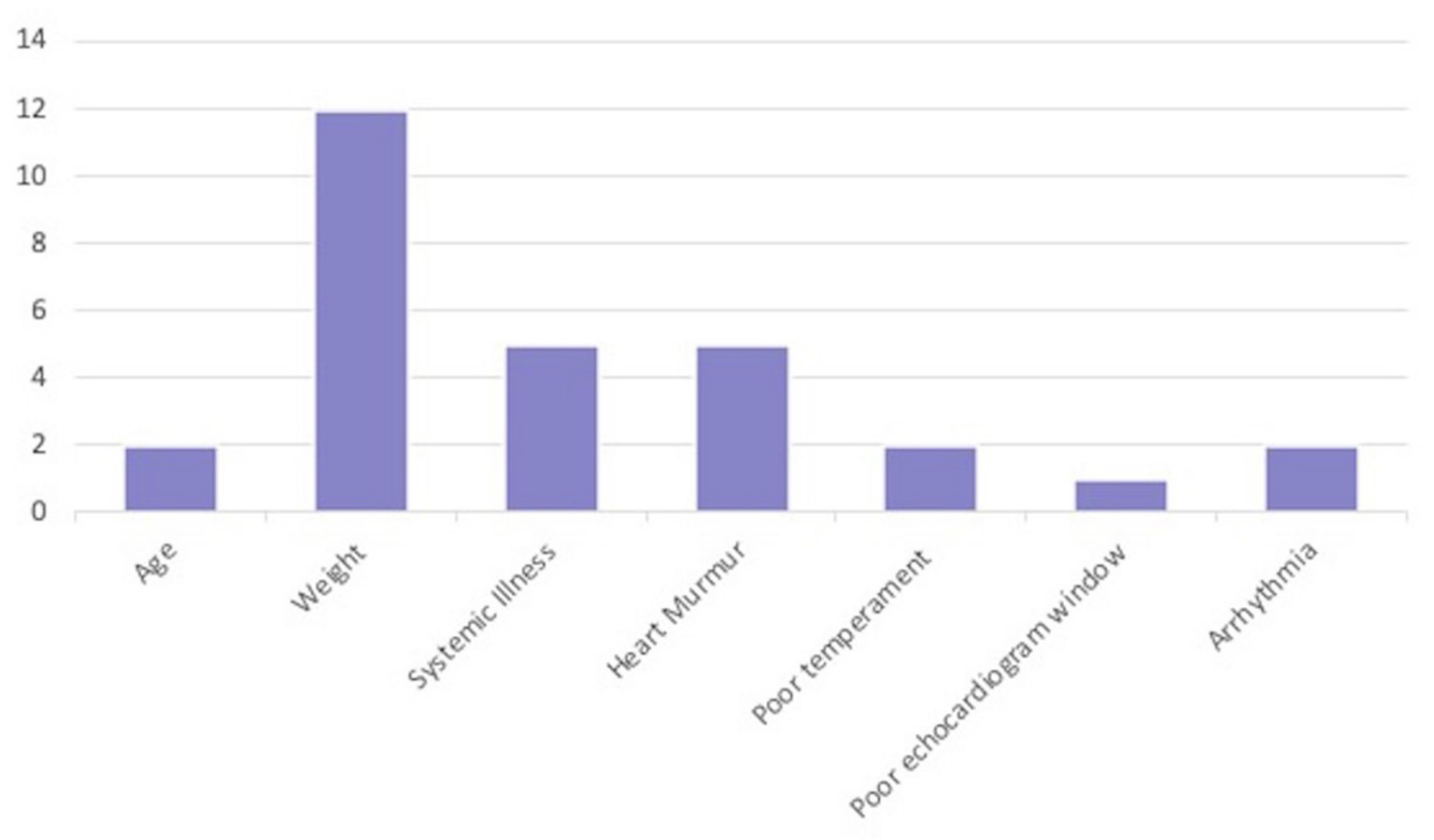
Specific Dog factors (*n* = 29) that led to exclusion in all three stages.

Of the 29 dogs excluded for Dog factors, the most common reason was not fitt ing within the weight range of the study (*n* = 12; 10 in Stage 1, 2 in Stage 2). The next most common Dog factor for exclusion was heart murmur > grade II/VI auscultated on physical examination (*n* = 5). Two dogs had arrhythmias detected on physical examination. A total of five dogs were also excluded for evidence of historical or current systemic illness including hypothyroidism on records review (*n* = 3), or clinical pathology (*n* = 2) (Table 1 and Figure 2).

## Discussion

Only 30% of dogs nominated by their owners for this RCT were subsequently enrolled. Stage 1 was the most common exclusion period (owner survey) (*n* = 19) with weight outside the target range being the most common reason for exclusion (*n* = 10 in Stage 1). Recruitment materials specified age and weight restrictions; therefore, it was unexpected that 18% of dogs were excluded based on their owner-reported weights. An additional two dogs' medical records revealed that the dogs weighed more than their owners reported. Dog factors (*n* = 29) for exclusion far outnumbered Owner factors (*n* = 6) for exclusion. In addition, cardiac abnormalities (*n* = 5 confirmed murmurs, *n* = 2 arrhythmias), and suspected or confirmed systemic illnesses (*n* = 7) were common exclusion factors. Overall, recruitment of healthy mature adult and senior dogs for this clinical trial was challenging, with 70% of all dogs recruited being excluded.

These enrollment challenges suggest a need to evaluate the criteria that define a mature adult or senior dog as having normal health status for its age. The authors propose that “normal health” should be reframed as “typical health” or “normative aging” when a mature adult or senior canine population is sought for study. In human health research, healthy cohort bias, also called healthy volunteer bias, exists when the population willing or able to participate in clinical research is in better health than the comparable demographic in the general population. In human studies, this phenomenon is often a consequence of individuals with better than average health being more likely to volunteer for and remain in longitudinal studies (46, 47, 50–52). Healthy cohort bias creates a risk that results of such studies will not be generalizable to the target population (53–55). To the authors' knowledge, the impact of healthy cohort bias has not been described in veterinary medical studies. Because owners rather than the dogs themselves volunteer for veterinary clinical trials, the factors known to precipitate healthy cohort bias in human studies may not apply, and it is even possible that some owners may fail to disclose certain illnesses in their dogs to try to ensure inclusion. However, healthy cohort bias could be created in veterinary clinical trials by use of inappropriate exclusion criteria in an aging population.

Cardiac abnormalities, including both cardiac arrhythmias and cardiac murmurs, led to the exclusion of seven dogs in Stage 3, which accounts for half of the total number of dogs excluded during Stage 3. Willems et al. (21) performed examination and diagnostic testing on 100 mature adult and senior dogs whose owners considered them to be healthy. A systolic heart murmur was detected in 22 dogs (22%) and the odds of murmur diagnosis significantly decreased with increasing weight (21). Similarly, five dogs in the present study were excluded for cardiac murmurs in Stage 3 representing 16% of all dogs examined in Stage 3. Although, a cardiac murmur is an indication of an increase in the turbulence of blood flow through the heart, such turbulence does not always lead to clinical consequences. Mitral valve regurgitation is a common (56) cause of a heart murmur in aging dogs that has limited clinical significance in many of those dogs. In one study, even when a murmur was not heard, mitral valve regurgitation from degenerative valve disease was identified by echocardiogram in more than 25% of dogs over 6 years old (41). In addition, over 60% of dogs that are asymptomatic for degenerative mitral valve disease will remain asymptomatic for years and are unlikely to die of cardiac related causes (57, 58). It may be appropriate to consider a heart murmur, and/or the finding of degenerative mitral valve disease, to be a typical part of the aging process in dogs. Degenerative mitral valve disease does represent an acquired change in cardiac performance. This matter is further complicated by the fact that degenerative mitral valve disease may have different pathologic significance in small dogs than in large dogs. But if the disorder occurs commonly, and often without associated morbidity, it may be appropriate to accept it as part of normative aging for some sizes and breeds of dogs. If so, then a trial enrolling normal mature adult or senior dogs should not exclude dogs of such sizes or breeds with degenerative mitral valve disease at the risk of creating healthy cohort bias.

Ventricular premature complexes (VPCs) led to the exclusion of two dogs in this study. There are a variety of cardiac and non-cardiac causes of VPCs in dogs, including splenic disease, dilated cardiomyopathy, anesthesia, and myocarditis (59). VPCs can also be sporadic without an underlying cause (60). A study using a 24-h ambulatory electrocardiogram in clinically normal Beagles aged 8–24 months found that VPCs were detected in 18.8 to 26.1% of the EKG analyses (60). Most VPCs were single and occurred sporadically, suggesting that sporadic VPCs throughout the day in dogs may not be clinically important. Since a single VPC captured during a 5-min EKG trace may be clinically insignificant, it may be necessary to use Holter monitors to better characterize VPC frequency among mature adult or senior dogs being screened for clinical trials. A better description of the likelihood of detecting sporadic VPCs in this population is also needed to determine if sporadic VPCs should be recognized as a typical part of normative aging.

Increase in liver enzyme (e.g., ALT or ALP) activity has been described as one of the most common bloodwork abnormalities in older dogs, with ALT and ALP enzyme activities increased in 25 and 27%, respectively, of mature adult and senior dogs (21). More than 50% of these increases were mild, being only 1–2 times the reference interval. Often times in clinical practice, these values in asymptomatic dogs do not prompt further investigation, especially if ALP enzyme activity is increased without an increase in ALT activity, unless these values become persistently elevated (22). In the present study, two dogs were excluded due to liver enzyme elevation. One dog had increases in both ALT and ALP activities. However, both of these values were <2 times the reference interval. The other dog had an increase in ALP activity alone, and this value was >3 times the reference interval. Based on owner-reported history and medical records review, these dogs had not been exposed to topical or systemic steroids. Clinically, both of these dogs were asymptomatic and had these values been found on an annual examination rather than a clinical trial screening assessment, many clinicians may not have pursued them further.

In human medicine, many expected physiologic and clinicopathologic changes with aging are well-defined, making it possible to identify a group of older patients as “normal” even if they exhibit some organ functional deterioration compared to younger people (61–66). By contrast, there is scant published information documenting expected age-related changes in dogs, and the ages at which such changes occur. It is likely that some similar changes in aging dogs are so consistently expected that they are, in fact, normal. While practitioners use their judgment to determine when such findings likely do not require extensive investigation, they have little data to guide them in this determination. Senile lenticular nuclear sclerosis is one of the best-known examples of such a finding, and is generally described as an expected and progressive change (67). It is one of the leading abnormal findings reported in Banfield's State of Health 2013 report, found in approximately 25% of dogs >10 years old (68). However, the frequency and age at onset of this change has not been prospectively described in a large companion dog population to the authors' knowledge. Better definitions of expected aging changes are needed.

Studies of pharmaceutical interventions are typically designed to test the ability to treat a specific disease, and focus on individuals with that disease. Modern aging research is now attempting to identify pharmaceutical treatments that prolong a healthy state, and as such, are focused on treating older, healthy, individuals (27, 28, 69–78). Companion dogs are ideal targets of healthy longevity research for two reasons. As an outbred species living in a variable environment, the companion dog serves as an excellent translational model species for humans (79–87). Additionally, dog owners and veterinarians seek to optimize canine healthy longevity because of the strong societal value placed on dogs, and the deep human-animal bond shared with them. As such, companion dogs are both participants in research into interventions to enhance their health and also the intended recipients of the benefits of that research (83, 88, 89). To successfully evaluate interventions to improve healthy lifespan in dogs, while avoiding healthy cohort bias, investigators must devise meaningful and realistic exclusion criteria for prospective studies. Future work is needed to define the expected frequency and severity of cardiac murmurs, singlet VPCs, increases in liver enzyme activity, and other common conditions associated with expected age-related decline among dogs. Until then, investigators must consider tolerating these apparently common conditions when devising exclusion criteria for studies of healthy mature adult and senior dogs. In the study reported here, 70% of candidate dogs were excluded for findings that may have been expected for their age and may have been of little clinical consequence. Studies of mature adult and senior dogs that exclude all dogs with any evidence of any disease may not yield results that can be generalized to all mature adult and senior dogs or translated to aging humans.

## Data Availability Statement

The raw data supporting the conclusions of this article will be made available by the authors, without undue reservation.

## Ethics Statement

The animal study was reviewed and approved by TAMU Institutional Animal Care and Use Committee (IACUC 2017-0125 CA). Written informed consent was obtained from the owners for the participation of their animals in this study.

## Author Contributions

HM devised the coding system for exclusion criteria and initiated data collection. NG completed data collection and analysis and contributed to writing the manuscript. DP, MK, and KC ran the clinical trial for which these dogs were recruited, supervised data analysis, and contributed to writing the manuscript. All authors contributed to the article and approved the submitted version.

## Conflict of Interest

The authors declare that the research was conducted in the absence of any commercial or financial relationships that could be construed as a potential conflict of interest.

## Abbreviations

ALT: Alanine aminotransferase
ALP: Alkaline phosphatase
CVD: Chronic valvular disease
ECG: Electrocardiogram
GFR: Glomerular filtration rate
RCT: Randomized controlled trial
S1: Stage 1
S2: Stage 2
S3: Stage 3
VPC: Ventricular premature complex.
